# Recent advances in the therapeutic potential of cathelicidins

**DOI:** 10.3389/fmicb.2024.1405760

**Published:** 2024-06-26

**Authors:** Maria Eduarda Souza Guerra, Brenda Vieira, Ana Paula Carvalho Thiers Calazans, Giulia Vicente Destro, Karina Melo, Emilly Rodrigues, Natalha Tedeschi Waz, Raquel Girardello, Michelle Darrieux, Thiago Rojas Converso

**Affiliations:** Laboratório de Biologia Molecular de Microrganismos, Universidade São Francisco, Bragança Paulista, Brazil

**Keywords:** inflammation, autoimmunity, antimicrobial peptides, cathelicidins, therapeutics, nanomedicines

## Abstract

The alarming increase in antimicrobial resistance in the last decades has prompted the search for alternatives to control infectious diseases. Antimicrobial peptides (AMPs) represent a heterogeneous class of molecules with ample antibacterial, antiviral, and antifungal effects. They can be found in many organisms, including all classes of vertebrates, providing a valuable source of new antimicrobial agents. The unique properties of AMPs make it harder for microbes develop resistance, while their immunomodulatory properties and target diversity reinforce their translational use in multiple diseases, from autoimmune disorders to different types of cancer. The latest years have witnessed a vast number of studies evaluating the use of AMPs in therapy, with many progressing to clinical trials. The present review explores the recent developments in the medicinal properties of cathelicidins, a vast family of AMPs with potent antimicrobial and immunomodulatory effects. Cathelicidins from several organisms have been tested in disease models of viral and bacterial infections, inflammatory diseases, and tumors, with encouraging results. Combining nanomaterials with active, natural antimicrobial peptides, including LL-37 and synthetic analogs like ceragenins, leads to the creation of innovative nanoagents with significant clinical promise. However, there are still important limitations, such as the toxicity of many cathelicidins to healthy host cells and low stability *in vivo*. The recent advances in nanomaterials and synthetic biology may help overcome the current limitations, enabling the use of cathelicidins in future therapeutics. Furthermore, a better understanding of the mechanisms of cathelicidin action *in vivo* and their synergy with other host molecules will contribute to the development of safer, highly effective therapies.

## Introduction

Antimicrobial resistance (AMR) presents a significant challenge to global public health, causing conventional antibiotics to be ineffective against many bacterial infections (Abushaheen et al., 2020). Prolonged and indiscriminate use of antibiotics has accelerated the emergence of multidrug-resistant strains, increasing mortality, morbidity, and healthcare costs (Bengtsson-Palme et al., 2018; Magana et al., 2020). The implications of AMR are profound, impacting both individual patients and global healthcare systems. Infections caused by drug-resistant pathogens are associated with higher rates of treatment failure, prolonged illness, and increased mortality. Additionally, the economic burden of AMR is substantial, including increased healthcare costs, prolonged hospitalizations, and reduced productivity.

The World Health Organization (WHO) estimates 1.3 million deaths globally due to antimicrobial resistance, with a projective annual death toll of 10 million by 2050 (World Health Organization, 2015). Furthermore, the global cost is expected to be around US$1.1 trillion by 2030 (World Bank, 2017). In 2015, the WHO released a Global Action Plan to Control Antimicrobial Resistance, including an increased investment in new medicines, diagnostic tools, vaccines and other interventions (World Health Organization, 2015).

The use of AMPs has emerged as promising alternative due to their unique properties and ability to overcome resistance mechanisms AMPs are part of the innate immunity in several organisms, aiding in the defense against pathogens. These peptides can be stored in neutrophil granules and macrophages, as part of the oxygen-independent bactericidal activity against pathogens. Their production can be constitutive or induced, varying according to the organism, peptides sequence, and cell type (Kosciuczuk et al., 2012; Dijksteel et al., 2021; Moretta et al., 2021).

AMPs display great variations in amino acid sequence and structure but share some features; most AMPs are small molecules—usually composed of 12 to 50 amino acids—rich in arginine and lysine residues, which confer a general positive charge, making them cationic. These chemical properties allow these molecules to easily disrupt and/or permeate the membrane of microorganisms, which have a negative charge, resulting in their death (Mookherjee et al., 2020). Besides interacting with charged membranes, AMPs display multiple antimicrobial and immunomodulatory properties. Many peptides can be translocated through membranes and bind to intracellular targets, modulating gene expression, protein synthesis and organelle function; others can bind to receptors in immune cells, mediating microbicidal, immunomodulatory or apoptotic responses (Scheenstra et al., 2020; Luo and Song, 2021; Bhattacharjya et al., 2024).

**Figure 1:**
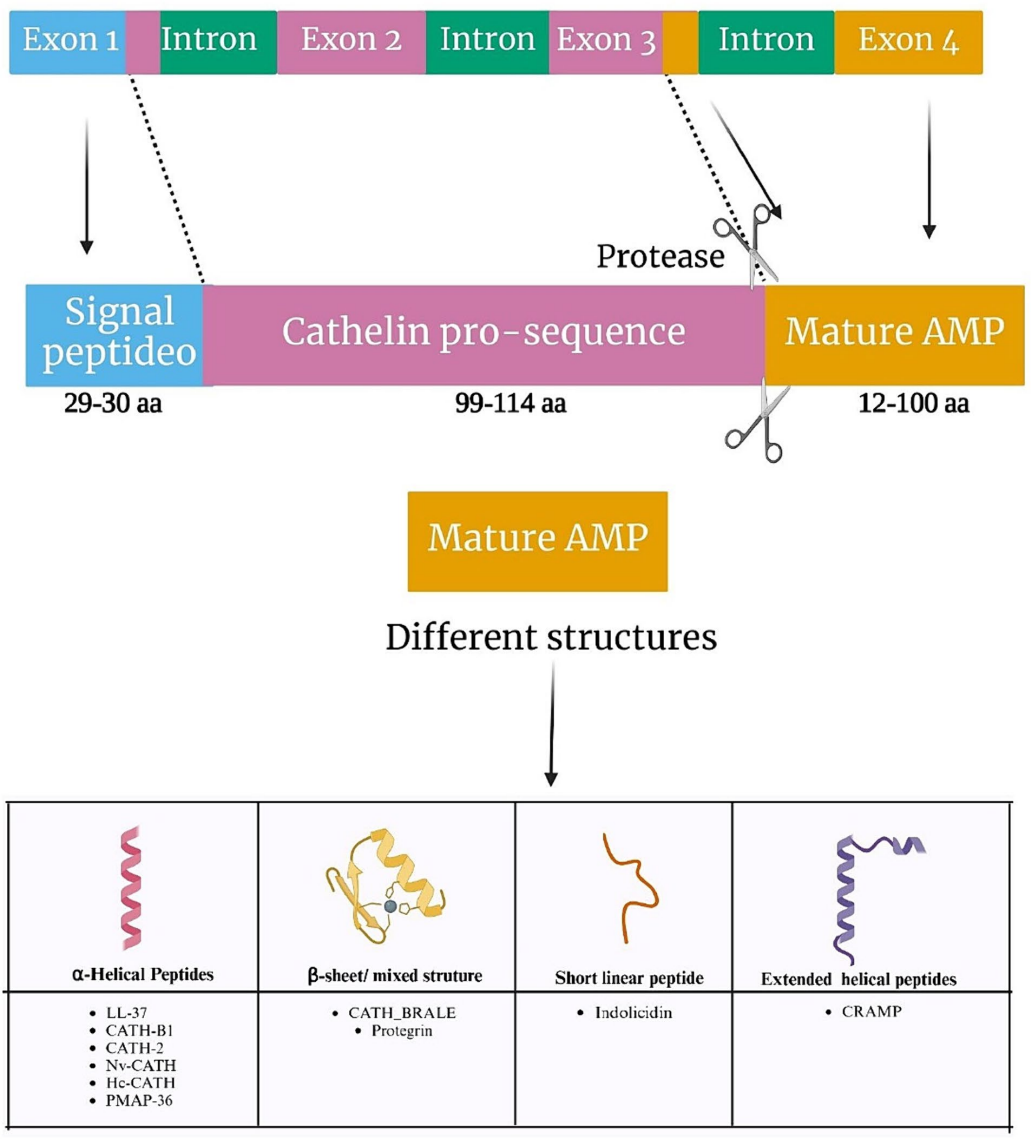
Genetic organization of cathelicidins. Schematic representation of the gene orientation of cathelicidins, with the respective cleavage site to produce the mature peptide (upper part), and the main types of cathelicidin structures (bottom part).

Cathelicidins represent a class of cationic antimicrobial peptides distributed across various organisms, including mammals, birds, reptiles, amphibians, and fish. Within mammals, the cathelicidin family encompasses approximately 30 peptides. The number of functional genes encoding cathelicidins varies among species, with humans, mice, rats, and dogs possessing a single encoded gene, whereas pigs, cows, rabbits, horses, goats, and sheep harbor up to 11 distinct cathelicidin genes. The genetic structure responsible for cathelicidin synthesis comprises four exons. Notably, exon 1 encodes a sequence spanning 29 to 30 amino acids, while exons 2 and 3 collectively encode a conserved domain, known as cathelin, consisting of 99 to 114 amino acids. This structural arrangement gives rise to the name “cathelicidins” (Figure 1; Kosciuczuk et al., 2012). Finally, exon 4 encodes the mature peptide, ranging from 12 to 100 amino acids, which exhibits antimicrobial and immunomodulatory activities (van Harten et al., 2018).

Many cathelicidins exhibit an unstructured conformation in aqueous solutions but adopt amphipathic α-helical structures in the presence of environments mimicking biological membranes. Others are larger molecules with repetitive proline-repeats. Cathelicidins also include a few small peptides sharing beta-hairpin structures stabilized by disulfide bonds, and one 13-residue peptide, indolicidin, presenting a linear structure with a high tryptophan content (Mhlongo et al., 2023). A common trait to all cathelicidins is the presence of a conserved N-terminal domain in the precursor protein called cathelin, which is cleaved to generate the active peptides (Figure 1; Mhlongo et al., 2023).

The ability to interact with microbial membranes is a consequence of the amphipatic nature of cathelicidins and directly linked to their microbicidal effects. Besides their membrane interacting effects, these molecules can permeate the membranes and bind to intracellular components like protein complexes, ribosomes, DNA, or RNA, affecting multiple processes, from protein synthesis and folding to peptidoglycan biosynthesis, respiration and detoxification of reactive oxygen species (Panteleev et al., 2018; Rowe-Magnus et al., 2019).

Collectively, the literature data suggest that cathelicidins perform several important functions for the host, contributing to the direct elimination of pathogens by their microbicidal action, as well as modulating host immune responses, wound healing, and tumor development. For their potent antimicrobial and immunomodulator activities, many cathelicidins from different sources have been investigated as therapeutic agents for infectious/autoimmune diseases and tumors. Notably, the field has witnessed the development of synthetic cathelicidins engineered to exhibit enhanced antimicrobial efficacy with reduced toxicity, an advancement that holds promise for large-scale, cost-effective production (Overhage et al., 2008; Boehmer et al., 2011; Nagaoka et al., 2020). The present review summarizes the latest studies investigating the therapeutic use of cathelicidins, their limitations and perspectives in the field (summarized in Table 1).

**Table 1:** List of the cathelicidins, their sources and therapeutic applications.

Cathelicidin	Source	Therapeutic applications	References
LL-37	Human	Antimicrobial, immune modulation, anti-tumoral effect; auto-immune diseases control	Niemirowicz et al. (2016), Shahmiri et al. (2016), van Harten et al. (2018), Matougui et al. (2019), Mookherjee et al. (2020), Nagaoka et al. (2020), Sancho-Vaello et al. (2020), Wang et al. (2021), Yu et al. (2021), Agadi et al. (2022), Duan et al. (2022), Lokhande et al. (2022), Waz et al. (2022), White (2022), and Yang et al. (2023)
CAT	Buffalo	Antimicrobial, immune modulation	Xu et al. (2019)
BMAP-28	Cattle	Antimicrobial, immune modulation	Mookherjee et al. (2020), Agadi et al. (2022), and Waz et al. (2022)
BMAP-27			
Indolicidin			
CATH-1	Chicken	Antimicrobial, immune modulation; anti-tumor effect	Peng et al. (2020) and Mahmoud et al. (2022)
CATH-B1			
CATH-H			
CAT_BRAILE	Fish	Antimicrobial, immune modulation; anti-tumoral effect; auto-immune diseases control	Chen et al. (2019)
CodCath1			
Nv-CATH	Frog	Antimicrobial, immune modulation	Shi et al. (2022)
CRAMP	Mouse	Antimicrobial, immune modulation; anti-tumoral effect	Wu et al. (2010), Umeshappa et al. (2021), Duan et al. (2022), and Yang et al. (2023)
PMAP-36	Pig	Antimicrobial, immune modulation	Agadi et al. (2022) and Waz et al. (2022)
Hc-CATH	Sea Snake	Antimicrobial, immune modulation	Liu et al. (2022) and Wang et al. (2022)
Ab-Cath	Snake	Antimicrobial, immune modulation	Qi et al. (2020) and Barman et al. (2023)
Bf-CATH			
Ovispirin	Sheep	Antimicrobial, immune modulation	Mookherjee et al. (2020)
B22	Synthetic	Antimicrobial, immune modulation; anti-tumoral effect; auto-immune diseases control	Kosciuczuk et al. (2012), Niemirowicz et al. (2016), Matougui et al. (2019), Rowe-Magnus et al. (2019), Browne et al. (2020), Qi et al. (2020), Wnorowska et al. (2020), Dijksteel et al. (2021), and Cristelo et al. (2023)
BF-30			
Ceragenin			
CSA-13			
CSA-131			
DPK-060			
LLKKK18			
Yongshi	Wild Boar	Antimicrobial, immune modulation	van Harten et al. (2018)
Protegrin	Yeast	Antimicrobial	Mookherjee et al. (2020)

## Antimicrobial properties of cathelicidins and therapeutic approaches

Cathelicidins display potent antimicrobial activity against Gram-positive and Gram-negative bacteria, performed by different mechanisms targeting an array of microbial components, including the bacterial membranes, cell wall components and multiple intracellular targets such as DNA, RNA, specific proteins and protein complexes (Raheem and Straus, 2019).

The main mechanism of antimicrobial action in cathelicidins involves interactions with the lipid bilayers that may lead to pore formation and/or membrane disruption (Neville et al., 2006). The classical models describing the interaction of AMPs with target membranes include transmembrane models, namely the barrel-stave and toroidal pore models, both resulting in holes in the membrane, and pore-less models, represented by the carpet/detergent-like model and the agglutination model (see Supplementary Figure 1; Mookherjee et al., 2020).

In the barrel-stave method, the CAMPs are inserted perpendicularly to the lipid bilayer, forming pores. The hydrophilic part of the AMPs interacts with the interior of the barrel, while the hydrophobic part will interact with the lipids within the membrane, facilitating the transport of substances, resembling a barrel. In the toroidal model, the peptides are adsorbed to the lipid bilayer at low concentrations, and at high concentrations, they insert vertically into the bilayer. In an assay mimicking a bacterial membrane, treatment with a bovine cathelicidin, BMAP-28 disrupted the packing of the bilayer, resulting in thinning of the membrane and pore formation (Agadi et al., 2022). This mechanism is shared among other members of the cathelicidin family with alpha-helical structures, such as LL-37, Dermcidin, BMAP-27, indolicidin and PMAP-36 (Agadi et al., 2022; Waz et al., 2022).

Conversely, in pore-less models, the CAMPs do not transverse the membrane but instead cover it, like a carpet. The interaction between the peptides and phospholipids generates disturbances in the bilayer, making the membrane more fluid. In an action analogous to that of detergents, the peptides form micelles with the lipids that impair the cell membrane function (Agadi et al., 2022).

Furthermore, the interaction between two different peptides appears to result in a greater membrane permeabilization capacity, possibly due to the formation of a more active complex on the membrane. This phenomenon has been demonstrated previously between peptides from different classes, such as defensins and human cathelicidin, and more recently, between two cathelicidins derived from *Cetartiodactyla* (Rashid et al., 2016).

Although the processes leading to membrane disruption by CAMPs are described independently, different mechanisms might be present for the same peptide. Furthermore, there are important differences in the interaction of cathelicidins with target membranes and novel mechanisms have been described (Brogden, 2005; Sevcsik et al., 2008; Shahmiri et al., 2016; Sancho-Vaello et al., 2020). For instance, LL-37 can act in two distinct ways: as a pore-former in unsaturated or cholesterol-containing lipids and as a membrane-modulating agent in saturated lipids, causing the formation of peptide-lipid tubular and fibrillar superstructures (Shahmiri et al., 2016; Sancho-Vaello et al., 2020). The study also suggests that mixed aggregation (formation of fibers) of LL-37 with lipids is a preferred route of action (Shahmiri et al., 2016). Oligomerization has been related to the formation of salt bridges during the peptide interaction with the membrane.

In addition, each cathelicidin may position itself differently in membranes. It has been demonstrated, for example, that Ovispirin and LL-37 are oriented parallel to the plane of the lipid bilayer, while Protegrin, isolated from yeast, appears to be inclined at a 55° angle relative to the surface of the bilayer (Mookherjee et al., 2020).

Permeability of the membrane and loss of bacterial shape are also described mechanisms of action. In an experimental imaging assay with *V. cholerae*, treatment with B22 (a cathelicidin derived from cattle) resulted in loss of shape and shorter cells within a few seconds, while control bacteria maintained their comma-shaped form, characteristic lengths, and membrane integrity (Rowe-Magnus et al., 2019).

Importantly, the described mechanisms of membrane disruption by CAMPs have been determined through *in vitro* experiments and may not accurately represent the conditions present during host infections.

One of the cathelicidins mechanisms of action is the pore formation and/or membrane solubilization, and it is not fully selective for microbial cells; as a result, they can cause toxic effects for host cells due to non-specific interaction with biological membranes. High therapeutic doses also present a challenge, triggering cytotoxic and/or hemolytic effects, limiting their use, which is why it is necessary to optimize these peptides (Ron-Doitch et al., 2016; Browne et al., 2020; Dijksteel et al., 2021).

To overcome the cytotoxicity problem, numerous investigations have demonstrated the potential of combining nanomaterials with active, natural antimicrobial peptides, including LL-37, and synthetic analogs like ceragenins. This has resulted in innovative nanoagents with significant clinical promise, capable of functioning as antimicrobial/anti-tumorigenic/regenerative agents, and immunomodulators (Kosciuczuk et al., 2012; Browne et al., 2020; Dijksteel et al., 2021). Ceragenins are compounds with chemical structure similar to AMPs, however it is based on cholic acid and its structural similarity allows ceragenins to preserve the broad-spectrum antimicrobial activity of AMPs, but their half-lives are not restricted by the action of proteases, and even long term storage in solutions does not affect their antimicrobial action (Wnorowska et al., 2020). In addition, the underling mechanism of action in ceragenins involves the electrostatic interaction between antimicrobial peptides and ceragenins with the anionic membranes of bacterial pathogens. This interaction facilitates rapid membrane insertion and depolarization leading to specific binding onto bacterial membrane and minimizing cytotoxicity (Wnorowska et al., 2020). Another advantage is that the ceragenins synthesis is cost efficient in comparison to relatively complex (~ 20–50 amino acids) AMPs considerably decreasing the production costs (this will be discussed further in the “Limitations” section).

Core-shell magnetic nanoparticles, integral to the realm of theranostics, boast a nuanced array of biological characteristics, making them a focal point in the quest for effective antibacterial strategies. Their capacity to disrupt bacterial cell membranes positions them as promising candidates for bactericidal action. A work by Niemirowicz et al. (2016) evaluated the synergistic interplay between core-shell magnetic nanoparticles and LL-37, against two important pathogens linked to antibiotic resistance, *Staphylococcus aureus* and *Pseudomonas aeruginosa*. Their findings unveiled a remarkable potentiation of antibacterial activity, when LL-37, the synthetic ceragenins CSA-13 and CSA-131 was paired with gold-coated core-shell magnetic nanoparticles where 64-fold and 32-fold decrease in the original MIC value of LL-37 peptide was observed (Niemirowicz et al., 2016). Such results underscore the potential of synergistic nanoparticle-peptide strategies in combating bacterial infections, providing better responses with lower AMP concentration, and contributing to reduce toxicity, heralding a promising avenue in immunological and microbiological for therapeutics research.

A novel study describes the versatility of lipid nano capsules (LNCs) as nanocarriers for therapeutic compounds, particularly antimicrobials, emphasizing their ability to adapt to a wide range of therapeutic molecules. LNCs have been explored for efficient delivery of hydrophilic molecules, including AMPs, through adsorption and encapsulation strategies (Matougui et al., 2019). The adsorption of AMPs onto the surface of LNCs was investigated, demonstrating notable efficiency, primarily due to electrostatic and hydrophobic interactions between the molecules. Additionally, a transacylation strategy was explored to strengthen the binding between AMPs and LNCs, with promising results especially for the peptide LL-37, which affected more Gram-negative bacteria, the formulation reduced 2 to 4-fold depending on the tested strain. The authors successfully used the strategy to incorporate the AMPs LL-37 and DPK-060 into LNCs, with high encapsulation efficiency (Matougui et al., 2019). These results feature the ability of LNCs to be tailored for efficient delivery of AMPs according to their structure and composition, improving the delivery of the peptides and reducing their toxicity.

In a recent investigation by Nagaoka et al. (2020), the potential therapeutic utility of LL-37 in the management of murine sepsis was explored. The study elucidated LL-37’s multifaceted protective properties within the context of septic mice, highlighting three crucial aspects of its action. Firstly, LL-37 demonstrated the capacity to enhance the survival of cecal ligation and puncture (CLP) mice by effectively suppressing macrophage pyroptosis, a process known to trigger the release of pro-inflammatory cytokines such as interleukin-1β (IL-1β), thereby exacerbating the inflammatory responses associated with sepsis. Secondly, LL-37 was shown to amplify the release of neutrophil extracellular traps (NETs), structures recognized for their potent bactericidal activity, consequently affording protection to mice against CLP-induced sepsis. Lastly, LL-37 exhibited the ability to stimulate neutrophils, prompting the release of antimicrobial microvesicles, or ectosomes, which contributed to an improvement in the pathological condition associated with sepsis (Nagaoka et al., 2020). These findings collectively underscore the multifaceted potential of LL-37 as a therapeutic candidate in the management of sepsis, providing valuable insights for future research and clinical applications.

Cathelicidins have also been explored as potential antiviral therapy. Hc-CATH, a cathelicidin derived from the sea snake *Hydrophis cyanocinctus*, has shown potent inhibitory activity against ZIKA-virus infection in a pregnant mouse model – an effect that resulted from a combination of direct disruption of the viral envelope by the peptide and downregulation of a kinase receptor, AXL, that mediates ZIKV infection (Liu et al., 2022; Wang et al., 2022). Mouse cathelicidins and LL-37 have also shown to reduce liver infection with the Enterovirus 71 (EV71), through a mechanism that included regulation of antiviral responses in host cells, especially inhibition of EV71-induced IL-6 production, contributing to immunomodulatory responses and inhibition of virus binding on the host cells, however the authors cannot exclude other virucidal mechanisms (Yu et al., 2021).

Human (LL-37) and mouse (CRAMP) cathelicidins have been investigated as treatment for viral cardiomyopathy caused by the non-enveloped virus Coxsackievirus B3 (CVB3); the peptides inhibited CVB3 replication in primary cardiomyocytes by activating the heat shock protein HSP60, which inhibited apoptosis and prevented exosome-mediated viral dissemination (Yang et al., 2023).

The use of LL-37 as treatment for COVID-19 is controversial. The peptide is elevated during SARS-CoV-2 infection and may contribute to hypercoagulation, through a mechanism involving endothelial cell dysfunction, inflammation, NET formation and platelet activation, which may promote thrombosis in COVID-19 patients (Duan et al., 2022). The link between LL-37 and hypercoagulation was also tested in mice injected with the cathelicidins LL-37 and CRAMP, which presented increased occurrence of thrombosis, whereas deletion of the cathelicidin gene inhibited thrombosis in the animals (Duan et al., 2022).

On the other hand, LL-37 has been shown to block the binding of SARS-COV-2 spike protein to the ACE2 receptor in host cells by an *in vitro* cell model and an *in vivo* mouse model of infection using a Pseudovirion. The authors show that LL-37 can bind to the RBD domains – which are responsible for the virus interaction with the host receptor – thus preventing the protein interaction with ACE2 and blocking viral entry (Wang et al., 2021).

Two other studies have predicted, *in silica*, that LL-37 can bind to the receptor-binding domain of SARS-CoV-2, preventing the virus interaction with host cells reinforcing the therapeutical potential of LL-37 against COVID-19 (Lokhande et al., 2022; White, 2022). In addition to LL-37, a screening of 16 cathelicidins found a peptide derived from wild boars, named Yongshi, that was able to reduce viral entry into host cells. The mechanism of action of this novel peptide involves an inhibition of the oligomerization of spike domains that allow the virus to enter the target cell; the AMP competes with one of the heptad repeats, inserting itself and disrupting the fusion process (van Harten et al., 2018). Interestingly, Yongshi was active against all variants of the virus tested, demonstrating a potential as a therapeutic agent against COVID-19 and opening a possibility to be studied against other viruses.

Clinical trials are underway to substantiate their efficacy, paving the way for their practical application (Browne et al., 2020). The combination of nanotechnology with the human cathelicidin, LL-37, continues to be an area of intensive study and exploration, aimed at developing new antibiotics with broad-spectrum antimicrobial activity and optimal bioavailability (Wnorowska et al., 2020).

**Figure 2:**
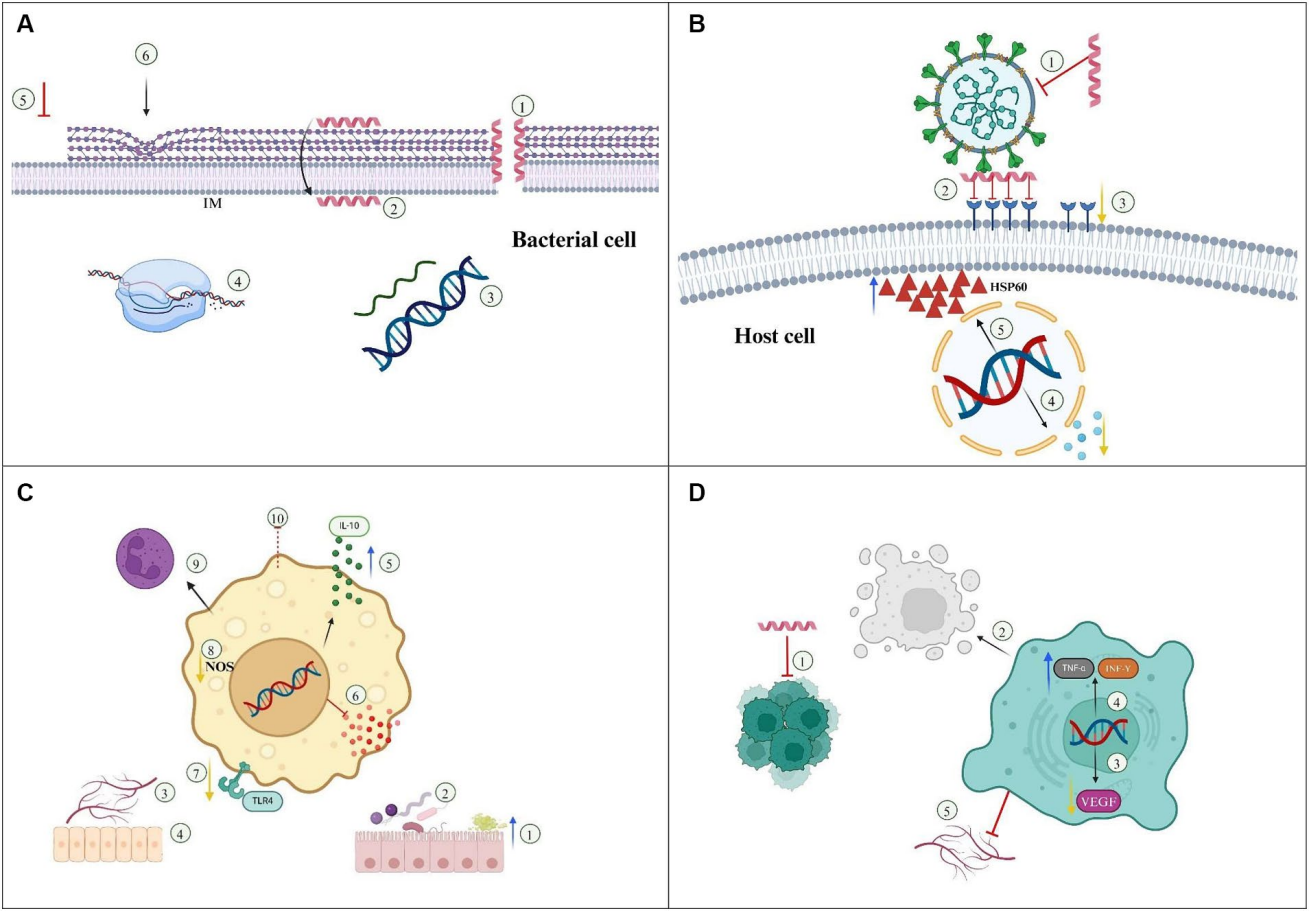
Mechanisms underlaying the therapeutic efficacy of cathelicidins/Nanoparticles. **(A)** Bacteria. 1—Membrane disruption, 2—Membrane translocation, 3—DNA/RNA synthesis, 4—Protein synthesis/folding, 5—Inhibition of peptidoglycan biosynthesis, 6—Alteration in cell shape. IM, Inner membrane. **(B)** Antiviral action. 1—Envelope disruption, 2—Inhibition of viral attachment/fusion/entry, 3—Down regulation of host receptors, 4—Down regulation of inflammatory cytokines, 5—Increased resistance to apoptosis by upregulation of HSP60. **(C)** Immunomodulatory /autoimmune diseases. 1—Increased mucus production, 2—Gut microbiota homeostasis, 3—Angiogenesis, 4—Reepithelization/wound healing, 5—Increased expression of IL-10, 6—Inhibition of pro-inflammatory cytokines, 7—Inhibition of TLR4 pathway, 8—Reduction in nitric oxide (NO) production, 9—Promotion of cell differentiation to anti-inflammatory phenotype, 10—Inhibition of cell trafficking. **(D)** Cancer treatment. 1—Inhibition of tumor cell proliferation, 2—Induction of apoptosis/necrosis, 3—Down regulation of VEGF, 4—Upregulation of TNF-α and IFN-γ, 5—Angiogenesis inhibition.

In summary, the recent advances in cathelicidin-mediated therapeutics against microbial infections suggest that these molecules are promising candidates for future antimicrobial agents, capable of controlling several types of bacterial and viral infections. Furthermore, the use of novel technologies such as nanoencapsulation and/or delivery systems potentiate antimicrobial efficacy, contributing to the development of cathelicidin-based therapies with reduced toxicity compared to the single AMP therapy. The therapeutic effects of cathelicidins against bacterial and viral infections are summarized in Figures 2A,B.

## Cathelicidins as therapeutics for inflammatory and autoimmune diseases

In addition to their antimicrobial action against multiple pathogens, cathelicidins play a pivotal role in modulating inflammation by influencing cytokine responses and the recruitment of inflammatory cells within diseased tissues. Besides the positive regulation of inflammatory responses associated with control of infectious diseases, newly published studies suggest that some cathelicidins display anti-inflammatory properties, indicating a possible therapeutic strategy for mitigating inflammatory diseases. The mechanisms of cathelicidin action against inflammatory/autoimmune diseases are summarized in Figure 2C.

A previous work by Wu et al. (2010) has explored the therapeutical used of cathelicidins in inflammation and tissue repair; the work highlighted the ability of a mouse cathelicidin, CRAMP, to reduce inflammation in the colon, primarily by boosting mucus production and concurrently diminishing the production of pro-inflammatory cytokines. Furthermore, cathelicidins have shown to promote wound healing, with their capacity to stimulate re-epithelialization and angiogenesis at sites of injury. Illustrating their versatility, a rat cathelicidin has been shown to promote ulcer healing in an animal model of gastric ulceration, inducing the proliferation of gastric epithelial cells both *in vitro* and *in vivo* (Wu et al., 2010). In summary, cathelicidins orchestrate a sophisticated interplay between inflammation and tissue repair in the gastrointestinal mucosa and various other organs, rendering them a promising target for therapeutic intervention.

A study by Peng et al. (2020) showed that a chicken cathelicidin, CATH-B1, downregulates gene expression of pro-inflammatory cytokine (IFN-β, IL-1β, IL-6 and IL-8) in primary macrophages, fostering an overall anti-inflammatory profile by significantly increasing IL-10 expression after avian pathogenic *E. coli* (APEC) challenge. Isothermal titration calorimetry (ITC) reveals that CATH-B1 binds to lipopolysaccharide (LPS), suggesting a reduction in toll-like receptor (TLR) 4-dependent activation by APEC, with a consequent decrease in production of pro-inflammatory cytokines by macrophages, suggesting a possible use as anti-inflammatory medicine (Peng et al., 2020).

An additional study discusses the identification of a novel 30-residue peptide, Nv-CATH, from the skin of the frog *Nanorana ventripunctata*, which belongs to the cathelicidin family. The authors have shown that Nv-CATH suppresses inflammatory responses by reducing the production of NO, IL-6, TNF-α, and IL-1β. The NF-κB-NLRP3 and MAPK inflammatory signaling pathways are implicated in this protective effect in both *in vitro* and *in vivo* settings. Nv-CATH also influences the trafficking of immune cells to the infection site and enhances immunocyte-mediated bacterial killing (Shi et al., 2022).

Looking at fish cathelicidins, a study by Chen et al. (2019) focuses on two fish-derived cathelicidins, CATH_BRALE and codCath1, which display potent immunomodulatory functions, inhibiting pro-inflammatory cytokine gene expression (TNF-α, IL-1β, and IL-6). This result suggests that CATH_BRALE and codCath1 inhibit the bacteria-induced hyperactivation of zebrafish innate immune system and excessive production of pro-inflammatory cytokines, resulting in controlled responses that protect against severe bacterial infections without harming the host. In addition, the study showed that both cathelicidins can stimulate the expression of the chemokine IL-8 in zebrafish., inducing the recruitment of monocytes, macrophages, and neutrophils to the infection site, which can kill the invading bacteria by phagocytosis.

Due to its ability to modulate the immune response, cathelicidins have also been investigated as therapeutics in autoimmune disease models. A recent study investigating the interplay between gut microbiota establishment and development of autoimmune, type I diabetes in neonate mice found that a defect in cathelicidin expression led to dysbiosis of the colon, which latter resulted in pancreatic autoimmune responses. The authors suggest that maintenance of cathelicidins production at steady levels in the gut is important for microbiota homeostasis, which may be used to prevent autoimmune diabetes in children at risk (Liang et al., 2022).

Another study used a cathelicidin derived peptide, LLKKK18, encased in a nanoparticle to treat type I diabetes in rats. The nanomedicine improved pancreatic function while reducing hyperglycemia, reinforcing the development of cathelicidin-based therapies for type-I diabetes (Cristelo et al., 2023). In addition to their protective role against autoimmune diabetes, mouse cathelicidins, CRAMP, have been implicated in activation of regulatory, type 2 neutrophils that prevent liver autoimmunity (Umeshappa et al., 2021).

A peptide derived from LL-37 was used as treatment in a murine model of collagen-induced rheumatoid arthritis (RA). Treated animals showed a reduction in inflammatory cells infiltrates in the joints and cartilage degradation, as well as improved clinical score, suggesting that synthetic cathelicidin analogs are an interesting approach to treat RA (Chow et al., 2014). Interestingly, this effect was restricted to the human-derived peptide.

A link between vitamin D levels and cathelicidin production has been suggested by different studies. Vitamin D can be obtained naturally through some dietary components or directly by the skin in presence of adequate sunlight. Immune cells such as dendritic cells and macrophages can actively metabolize the precursor 25-hydroxyvitamin D (25D) into active 1,25D during infections, which stimulates the expression of antimicrobial peptides like cathelicidin (Mercola et al., 2020; Bishop et al., 2021).

Aloul et al. has unveiled the correlation between vitamin D levels and LL-37 production in COVID-19 patients. The researchers emphasized that the higher expression of LL-37 can serve a therapeutic role in COVID-19 patients through different mechanisms, including an efficient clearance by neutrophil extracellular traps (NETs) and macrophages, endothelial repair following inflammatory tissue damage, prevention of α-synuclein aggregation, and stabilization of blood glucose levels by facilitating insulin release and islet β-cell neogenesis. All those mechanisms contribute to reduce the infection severity. Thus, the authors propose that vitamin D uptake can improve the outcome in COVID patients by promoting the production of LL-37 (Aloul et al., 2022).

In a recent study, Gubatan et al. have analyzed serum and colonic cathelicidin levels in ulcerative colitis patients and found that patients with higher vitamin D also expressed higher LL-37 levels, which correlated with decreased risk of histologic inflammation. The authors have also shown that treatment with Vitamin D stimulated cathelicidin production by human colon cells (Gubatan et al., 2020).

A link between vitamin D-regulated cathelicidin expression and Chron’s disease (CD) has been proposed by Vaccaro et al. (2022) using macrophages infected with *Mycobacterium avium* isolated from CD patients. The authors found that the infection disrupted vitamin D metabolism, leading to reduced levels of the AMP and worsening the disease (Vaccaro et al., 2022). The results suggest that cathelicidin production improves the prognosis of Chron’s disease patients; therefore, supplementation with cathelicidins may be used in future therapies to control the disease.

These findings contribute to understanding the immunomodulatory effects of cathelicidins, which can be explored as therapeutic strategies for treating inflammatory/autoimmune diseases; however, since cathelicidins present some limitations (see section above), more studies are needed to overcome these issues.

## Cathelicidins and cancer therapy

Cathelicidins from different organisms have been investigated as anti-tumor agents in several types of cancer, based on their immunomodulatory properties, interference with multiple cellular functions and the ability to disrupt and/or penetrate membranes, promoting cancer cell lysis or acting as chemotherapy carriers (Baxter et al., 2017). The mechanisms involved in the anti-tumor activity of cathelicidins are shown in Figure 2D.

CAT, a cathelicidin from buffalo, was evaluated as a delivery system for antitumor drugs in different cancer lineages, having shown high cell penetrating efficacy and increased apoptosis of target cells. This effect was more pronounced in human hepatoma cells, suggesting that the conjugation of CAT and cytotoxic agents is a promising therapeutic approach against tumors (Xu et al., 2019).

Peptides derived from snake venoms have been successfully evaluated against a range of tumor cells, including leukemia, liver, breast, and prostate cancers (reviewed in Perez-Peinado et al., 2020). Crotalicidin, a 34 aminoacid peptide isolated from the venom of a South American rattlesnake, has shown potent *in vitro* activity against leukemia cell lines, while topo isomers of the peptide’s C-terminal fragment displayed increased toxicity against tumor cells, especially leukemia and neuroblastoma cells lines (Perez-Peinado et al., 2020; Carrera-Aubesart et al., 2022). Nevertheless, they also showed increased toxicity to normal cells, suggesting that further studies are needed to produce modified molecules with high anti-tumor efficacy and selectivity to subvert the cytotoxicity.

A modified peptide named BF-30, derived from the snake *Bungarus fasciatus*, Bf-CATH, was produced by fusing heptapeptide-palmitic tags to the native peptide via a protease-cleavable linker and prepared by F-moc solid-phase synthesis. BF-30 inhibited tumor proliferation and angiogenesis in a mouse model of melanoma, through a mechanism involving peptide interaction with tumor cell DNA and inhibition of vascular endothelial growth factor expression. The molecule was also tested in a mouse cancer model, showing a significant suppression in melanoma growth and improved survival rates of B16F10-bearing mice. Cytotoxicity was analyzed *in vivo* in rhesus monkeys and showed that the modified version was less toxic and presented an increased half-live in comparison with the native AMP (Qi et al., 2020).

Chicken cathelicidins have also been evaluated as potential anti-tumor therapeutics *in vitro* and in mouse models. One peptide, CATH-1, was able to decrease tumor size, which correlated with increased production of TNFα and INF-γ, caspase activation and tumor necrosis (Mahmoud et al., 2022).

The effects of chemotherapy with LL-37 have been investigated in primary glioblastoma (GB) cell lines; treatment with the peptide alone showed high cytotoxicity to cancer cells, while the combination with chemotherapeutics [temozolomide (TMZ), doxorubicin (DOX), carboplatin (CB), cisplatin (CPL), and etoposide (ETO)] resulted in a synergistic effect. In addition to the encouraging results *in vitro*, administrations of LL-37 was able to inhibit tumor growth in a rat model of intracerebral GB, suggesting that the human cathelicidin has important anticancer activities that can be explored for the development of new therapeutic approaches against aggressive tumors (Chernov et al., 2024).

Finally, nanoparticles containing LL-37 encapsulated with siRNA against VAV1 (a protein that is overexpressed in many tumors and associated with metastatic dissemination and poor prognosis) was highly effective at suppressing tumor development in a mouse model of pancreatic ductal adenocarcinoma, increasing survival while reducing VAV expression. LL-37 served a dual role as both a counter ion for the negatively charged siRNA and an anticancer peptide (Agbaria et al., 2023). This approach emphasizes the potential of combining peptides with other chemotherapy drugs in nanomedicines to promote strong anticancer effects.

## Limitations in the therapeutic use of cathelicidins

Although antimicrobial peptides (AMPs) hold promises as therapeutic agents, their clinical application faces several immunological challenges. These limitations stem from issues such as cytotoxicity, biological instability, size constraints, high synthesis costs, immunogenicity, and hemolytic activity (Oliveira et al., 2020; van der Weide et al., 2020; Dlozi et al., 2022). Specifically, cathelicidins in general exhibit low bioavailability and are susceptible to degradation by proteases, thereby restricting feasible routes of administration. Moreover, their pleiotropic nature complicates systemic interventions, requiring precise therapeutic ranges to avoid undesired immunomodulatory effects (Zhang et al., 2023).

To surmount these challenges and enhance the efficacy of AMPs, innovative strategies involving bioinformatics tools such as sequence alignment and molecular docking for molecular interactions prediction, coupled with protein engineering techniques are being explored. These approaches aim to modify AMPs to enhance stability, specificity, size, and activity (Oliveira et al., 2020; Mousavi Maleki et al., 2021). Additionally, encapsulation strategies offer a promising avenue for stabilizing unstable peptides, ensuring target specificity, and preventing off-target effects on unaffected cells (Dlozi et al., 2022). Various delivery vehicles, including metallic nanoparticles, polymeric materials, and lipid-based systems, are under investigation. However, understanding the mechanisms underlying delivery systems, the biological performance of AMPs like LL37, and bond-induced conformational changes remains a challenge for clinical translation. While *in vitro* studies provide valuable insights, further *in vivo* validation is imperative to substantiate findings and facilitate clinical utilization in the field of immunology (Xiaoxuan Lin and Mai, 2020).

An example of peptide modification to overcome the disadvantages of limited bioavailability, cytotoxicity and stability is the use of nanogels. A study using hyaluronic acid (HA) modified with a long lipid chain (18 carbons) increased the permeability and fluidity of two peptides, one of them being a snake-derived catheleccin, Ab-Cath. The *in vitro* assays showed that the encapsulation maintained the antimicrobial properties, reduced hemolytic and cytotoxic activity, in addition, this formulation increased the level of selectivity of Ab-Cath by 16.8 times, revealing a great clinical potential (Barman et al., 2023).

In addition to these hurdles, the scalability and cost-effectiveness of production methods are crucial considerations for widespread commercial and clinical adoption. Current production approaches, including chemical synthesis and recombinant techniques involving bacteria, yeast, transgenic plants, and mammalian cells, incur significant expenses due to the complexity of AMP structures and the need for suitable expression systems. While various methodologies have been proposed, further investigation is required to determine the optimal cost–benefit solution (Pachon-Ibanez et al., 2017). To produce AMPS by solid-phase synthesis it costs $50–400 per gram of amino acid, while to produce aminoglycosides it costs $0.80 per gram. The synthesis of ultrashort and truncated peptides is a strategy sought by industries because it reduces their production cost; meanwhile, the use of encapsulation systems such as peptide delivery and chemical modifications improve bioavailability and reduce toxicity. Finally, combined therapy with antibiotics can improve the efficacy of the peptide and decrease cytotoxicity (Dijksteel et al., 2021).

Therapeutics based on peptides are being developed, which already occupy 5% of the global pharmaceutical market and in 2019 exceeded $50 billion in global sales. Peptide-based medicines in recent decades have been approved steadily and with an average growth rate of 7.7% for the global peptide therapeutics market (Barman et al., 2023).

Another point of concern is the use of human peptides in therapeutics, which could lead to development of resistance against natural host immunity. Although resistance to AMPs is much less common, it can still occur. The majority of clinical trials employing AMPs as therapeutics are based on human molecules (Lazzaro et al., 2020). An alternative to reduce this associated risk is to broaden the studies using peptides from other sources.

Key insights gleaned from these setbacks include the need to expand clinical indications, optimize dosag‑e and administration schedules, establish equivalence or non-inferiority to existing antibiotics, anticipate the development of bacterial resistance, and explore synergistic combinations with other compounds. By integrating these lessons into future clinical trials, the likelihood of success for peptide-based immunotherapies can be significantly enhanced.

## Conclusion

Several AMPs have been studied as alternative treatment against an array of diseases; projections for the global market of peptide therapeutics predict a record value of USD 44.43 billion in 2026. Currently, more than 80 peptide-based drugs are present in the market for the treatment of a wide range of diseases including cancer, osteoporosis, diabetes, etc. (Tripathi and Vishwanatha, 2022).

In the dynamic landscape of therapeutic innovation, cathelicidins have emerged as a captivating area of exploration, offering profound potential in the battle against microbial resistance. Their intrinsic antibacterial properties, coupled with their unique mechanisms that deter microbial resistance, have garnered considerable attention within the scientific community. Notably, advancements in synthetic cathelicidins engineered for heightened antimicrobial efficacy indicate a significant stride toward large-scale, cost-effective production. Despite promising developments, the clinical translation of peptide-based therapies faces multifaceted challenges, ranging from cytotoxicity to high production costs and issues surrounding bioavailability and efficacy. Several peptides have faltered in clinical trials, revealing the complexity inherent in surpassing the efficacy of conventional antibiotics. However, these setbacks provide invaluable insights for future endeavors. Strategies encompassing expanded clinical indications, optimized dosing regimens, and consideration of bacterial resistance dynamics offer promising avenues for further exploration. Moreover, the exploration of synergistic combinations with other compounds holds potential for enhancing the efficacy of peptide-based immunotherapies. By assimilating these lessons into future research and clinical trials, the trajectory of peptide-based therapeutics can be refined, potentially revolutionizing the landscape of antimicrobial interventions, and bolstering our armamentarium against microbial threats.

## Author contributions

MG: Data curation, Investigation, Methodology, Writing – original draft. BV: Investigation, Writing – original draft. GD: Investigation, Writing – original draft. KM: Investigation, Writing – original draft. ER: Investigation, Writing – original draft. NW: Investigation, Writing – original draft. RG: Writing – original draft, Writing – review & editing. MD: Writing – original draft, Writing – review & editing. TC: Conceptualization, Data curation, Funding acquisition, Supervision, Writing – original draft, Writing – review & editing. AC: Investigation, Writing – review & editing.

## Supplementary material

SUPPLEMENTARY FIGURE 1 Mechanisms of cathelicidin action on membranes. Cathelicidins can present 4 different mechanisms of action on the membrane: Translocation causing intracellular interactions such as DNA/RNA synthesis, and protein synthesis and/or folding, and mechanisms of membrane disruption such as Barrel Stave, Toroidal pore, and Carpe Model.
