# Supplementary figures and images for: Recent advances in the therapeutic potential of cathelicidins
Source: Front Microbiol. 2024 Jun 26;15:1405760. doi: 10.3389/fmicb.2024.1405760 (PMC11233757; doi:10.3389/fmicb.2024.1405760)

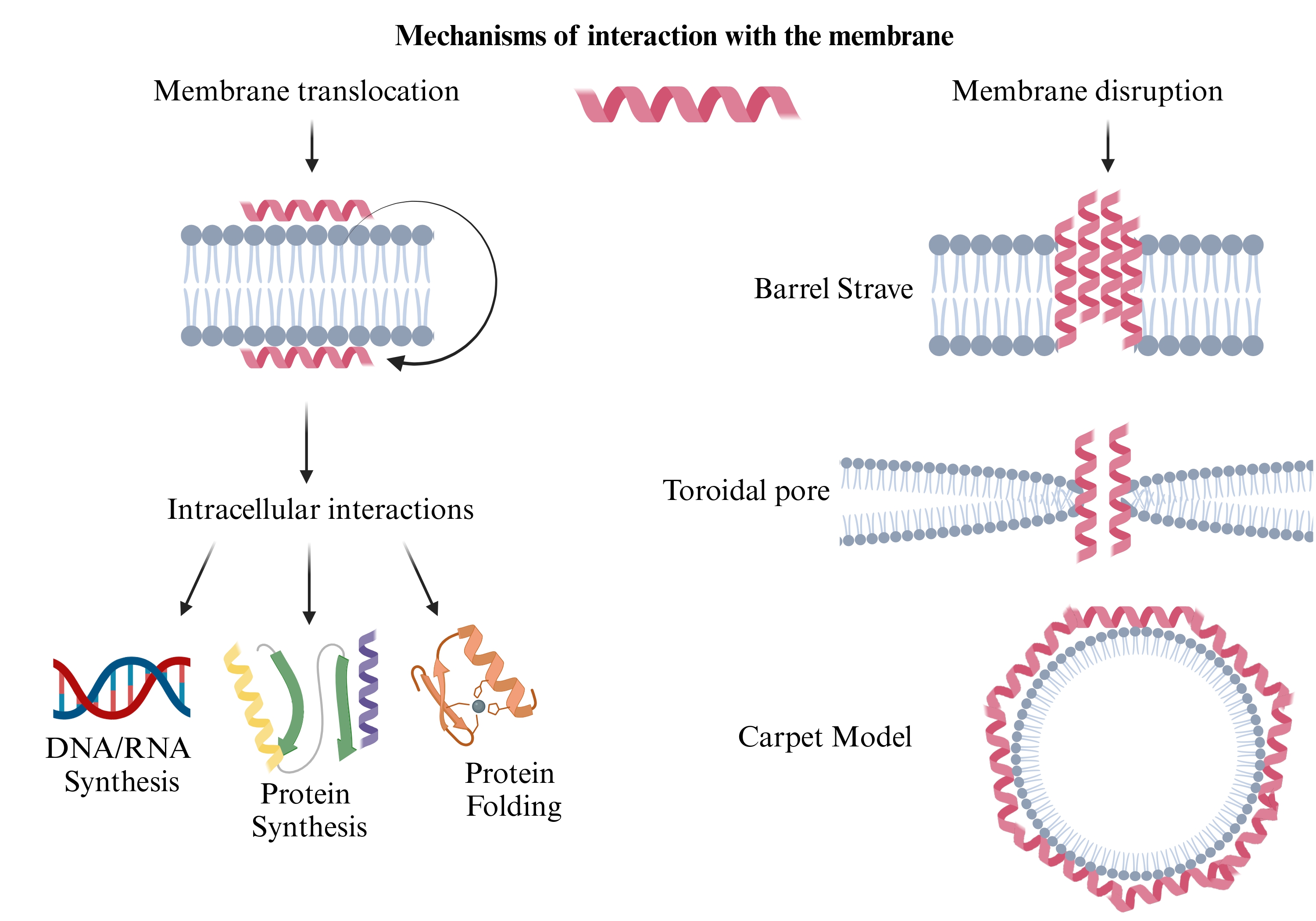

Supplement: SUPPLEMENTARY FIGURE 1 — Mechanisms of cathelicidin action on membranes. Cathelicidins can present 4 different mechanisms of action on the membrane: Translocation causing intracellular interactions such as DNA/RNA synthesis, and protein synthesis and/or folding, and mechanisms of membrane disruption such as Barrel Stave, Toroidal pore, and Carpe Model. [file Image_1.JPEG]
